# Patient and Family Involvement in Serious Incident Investigations From the Perspectives of Key Stakeholders: A Review of the Qualitative Evidence

**DOI:** 10.1097/PTS.0000000000001054

**Published:** 2022-08-02

**Authors:** Lauren Ramsey, Siobhan McHugh, Ruth Simms-Ellis, Kayley Perfetto, Jane K. O’Hara

**Affiliations:** From the ∗ Yorkshire Quality and Safety Research Group, Bradford Institute for Health Research, Temple Bank House, Bradford Royal Infirmary Duckworth Lane, Bradford, United Kingdom; † Queen’s University, Kingston, Canada; ‡ University of Leeds School of Healthcare, 3 Beech Grove Terrace, Woodhouse, Leeds, United Kingdom.

**Keywords:** patient safety, patient involvement, safety events, serious incidents, healthcare harm, patient experience, healthcare quality, healthcare, qualitative research, scoping review, review

## Abstract

Investigations of healthcare harm often overlook the valuable insights of patients and families. Our review aimed to explore the perspectives of key stakeholders when patients and families were involved in serious incident investigations.

**Methods:**

The authors searched three databases (Medline, PsycInfo, and CINAHL) and Connected Papers software for qualitative studies in which patients and families were involved in serious incident investigations until no new articles were found.

**Results:**

Twenty-seven papers were eligible. The perspectives of patients and families, healthcare professionals, nonclinical staff, and legal staff were sought across acute, mental health and maternity settings. Most patients and families valued being involved; however, it was important that investigations were flexible and sensitive to both clinical and emotional aspects of care to avoid compounding harm. This included the following: early active listening with empathy for trauma, sincere and timely apology, fostering trust and transparency, making realistic timelines clear, and establishing effective nonadversarial communication. Most staff perceived that patient and family involvement could improve investigation quality, promote an open culture, and help ensure future safety. However, it was made difficult when multidisciplinary input was absent, workload and staff turnover were high, training and support needs were unmet, and fears surrounded litigation. Potential solutions included enhancing the clarity of roles and responsibilities, adequately training staff, and providing long and short-term support to stakeholders.

**Conclusions:**

Our review provides insights to ensure patient and family involvement in serious incident investigations considers both clinical and emotional aspects of care, is meaningful for all key stakeholders, and avoids compounding harm. However, significant gaps in the literature remain.

Patient safety remains a persistent worldwide healthcare issue, despite commitment to improve since *To Err Is Human.*^1^ This is exemplified by widespread scandals of poor care quality^2,3^; however, incidents are also a longstanding focus within routine care. The United Kingdom alone reports more than 1 million safety-related events annually, including an estimated 10,000 defined “serious incidents,” which result in severe harm or death, costing approximately £1.7 billion in clinical negligence claims.^4^ Serious incidents can be defined as: “… events in health care where the potential for learning is so great, or the consequences to patients, families and carers, staff or organizations are so significant, that they warrant using additional resources to mount a comprehensive response” (National Health Service [NHS] England, Serious Incident Framework).^5^ Like all safety critical industries, healthcare systems are complex, adaptive, and dynamic, requiring an understanding of human factors to enable learning.^6^ Nevertheless, systems are arguably designed to detect “reckless” staff within the context of blame culture, overlooking valuable opportunities to ultimately make patients safer.^7,8^ The Kirkup report summarized that, “Errors occur in every healthcare system. What is inexcusable, however, is the repeated failure to examine adverse events properly, to be open and honest with those who suffered, and to learn so as to prevent recurrence.”^2^

Over the last decade, the importance of disclosing and investigating incidents has been recognized, as well as the potential for these processes themselves, to negatively affect stakeholders over and above incidents. In the United Kingdom, the Duty of Candor was introduced in 2014, meaning that healthcare professionals have since been legally obliged to be open and honest with patients when something has gone wrong that could, or has the potential, to cause harm or distress. Those deemed “serious incidents” have been investigated as per the Serious Incident Framework published by NHS England in 2015. However, one key stakeholder group often overlooked within safety investigations is patients and their families, despite their involvement considered both a moral obligation of health services^9^ and a valuable source of information.^10,11^ Investigations relying solely on staff reports and clinical notes often fail to access these perspectives^12^ as patients and families tend to be the only common denominator across health service interactions, including transitions through services, settings, and experiences with professionals over time.^13–15^ These unique insights have been argued to support both incident analysis and recommendations after healthcare harm,^16^ and NHS Resolution has recently posited that involving patients and families earlier in investigations may divert and reduce the cost of litigation.

Our review aimed to explore the current evidence surrounding the involvement of patients and families in serious incident investigations. Previous reviews have considered the open disclosure of adverse events^17,18^ and patients’ perspectives of incidents in hospital.^19^ However, in this review, we aimed to specifically address the following research questions:

1. What are the experiences, values, and needs of patients and families involved in serious incident investigations?
2. What are the experiences, benefits, and challenges from the perspectives of key stakeholders when patients and families are involved in serious incident investigations?
3. What potential solutions does the literature present to support designers of incident investigation systems support greater involvement?

## METHODS

A scoping review of the qualitative evidence was considered the most suitable approach to explore and summarize the emerging evidence. The Preferred Reporting Items for Systematic Reviews and Meta-Analyses Extension for Scoping Reviews guidance was followed.^20^ The search strategy was iteratively developed in collaboration with the project steering group and patient and family advisory group. The search comprised 4 search strings combined with AND relating to (1) population, that is, patients and families, and 3 separate strings relating to elements of the concept, that is, (2) serious incidents, (3) investigations, and (4) involvement (each combined with OR). The search was applied to 3 databases (OVID Medline [1946–present], APA PsychInfo 1806–present, and CINAHL) by one author (S.M.). Articles were imported to EndNote, and duplicates were removed before screening. The “population-concept-context” method was used to develop the eligibility criteria, which were iteratively refined by all authors (Table 1).

**TABLE 1:** Eligibility Criteria

	Eligibility Criteria
Population	Patients or their families. Articles may explore the perspective any stakeholder(s) regarding the involvement of this population.
Concept	Patient or family involvement in serious incident investigations, including investigation components (e.g., disclosure or reconciliation), or investigations in their entirety. Articles may use terms synonymous with serious incidents (e.g., adverse events, medical error) or focus on specific incident types (e.g., wrong site surgery, suicide). Related studies, but where the concept of interest was not the core focus, were excluded.
Context	Secondary care settings including acute care, mental healthcare or maternity in any country. Studies conducted in purely primary or community care or where death occurred outside of the healthcare setting or was deemed unrelated to a serious incident were excluded.
Study design	Any empirical research using qualitative methods, including secondary analysis of qualitative data or mixed methods research with a significant qualitative focus. Quantitative research, mixed methods research without a significant qualitative focus, study protocols, literature reviews and gray literature were excluded.
Other	Papers published in the English language post-2000, when patient safety arguably became a studied phenomenon.

Title and abstract screening was equally and independently conducted by 2 authors (S.M., K.P.), who peer checked 20% of each other’s decisions. Full-text screening was conducted collaboratively between 2 authors via discussion until a consensus was reached (S.M., R.S.E.). One author (K.P.) peer checked 20% of decisions with 100% agreeability (*K* = 1). All eligible articles were assessed using Connected Papers (http://www.connectedpapers.com/), an online tool that generates relevant articles based on cocitation and bibliographic coupling, by one author (L.R.) until no new articles were found. One author (K.P.) peer checked 20% of decisions.

**FIGURE 1:**
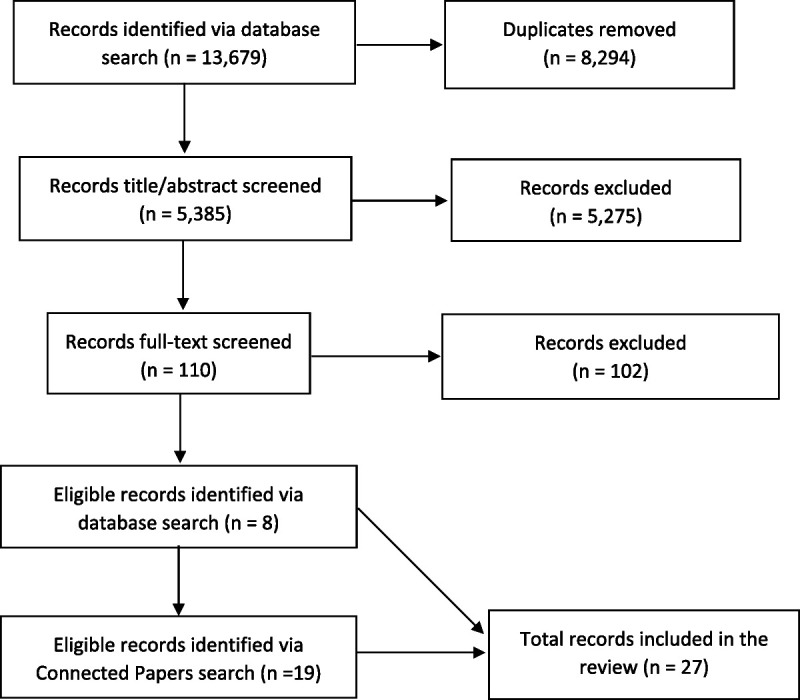
Preferred Reporting Items for Systematic Reviews and Meta-Analyses flow diagram.

A total of 27 articles were included in the review (Fig. 1). Data were extracted from eligible articles by 2 authors (L.R., K.P.) according to 3 broad categories comprising: study details (e.g., year, country, study aim), population (e.g., setting, sample), and study design (e.g., data collection, analysis, findings). Studies were not quality assessed as the core aim was to summarize emerging evidence, rather than compare individual study quality or assess the overall strength of a body of evidence. The extracted data were summarized via narrative synthesis,^21,22^ of which all authors discussed, contributed to, and refined.

## RESULTS

### Study Characteristics

Twenty-seven eligible articles were reviewed, published from 2003 to 2020, and based in the United States (n = 13, 48.1%), Australia (n = 6, 22.2%), the United Kingdom (n = 3, 11.1%), the Netherlands (n = 2, 7.4%), Norway (n = 2, 7.4%), and New Zealand (n = 1, 3.7%).

### Setting

Most focused on involvement in investigations across general hospital care settings (n = 22, 80.8%).^23–44^ Incidents predominantly related to acute services, but spanned primary, secondary, and tertiary care. Others specifically focused on antenatal or neonatal death in maternity or pediatric intensive care units (n = 4, 15.4%)^45–48^ and suicide in mental healthcare (n = 1, 3.8%^49^; Table 2).

**TABLE 2:** Study Characteristics According to Setting

Setting	Total Studies	Avg. No. Perspectives Sought Per Study	Total Perspectives Sought Across Studies	Total Perspectives Omitted Across Studies	Predominant Element(s) of Investigations Considered	No. Intervention Studies
General hospital care	22	1.8	4 (patient/family, healthcare professional, nonclinical, legal)	0	Disclosure element only (focus of 50% of studies)	16
Maternity	4	1	2 (patient/family, healthcare professional)	2 (nonclinical and legal)	Reconciliation element only (focus of 100% of studies)	0
Mental health	1	3	3 (patient/family, healthcare professional and nonclinical)	1 (legal)	Reconciliation element only (focus of 100% of studies)	0

### Focus of Intervention

Sixteen studies (59.3%) evaluated interventions supporting patient and family involvement comprising 6 intervention types, each based in general hospital care. These are categorized according to element(s) of the investigation targeted (Table 3).

**TABLE 3:** Study Characteristics According to Intervention

Intervention Type	Country	Total No. Studies	Avg. No. Perspectives Sought Per Study	Total Perspectives Sought Across Studies*	Total Perspectives Omitted Across Studies*	Predominant Focus
Open disclosure	Australia and United Kingdom	7	1.6	3 (patient/family, healthcare professionals, nonclinical)	1 (legal)	Disclosure only
CRPs	United States	4	2.3	4 (patient/family, healthcare professionals, nonclinical, legal)	0	Disclosure and reconciliation
Next-of-kin	Norway	2	1	2 (patient/family, nonclinical)	2 (healthcare professionals, legal)	Disclosure and reconciliation
DA&O	United States	1	3	3 (healthcare professionals, nonclinical, legal)	1 (patient/family)	Disclosure and reconciliation
3Rs	United States	1	1	1 (patient/family)	3 (healthcare professionals, nonclinical, legal)	Disclosure and reconciliation
IMPACT	United States	1	1	1 (patient/family)	3 (healthcare professionals, nonclinical, legal)	Reconciliation only

### Disclosure

Of the intervention studies, 7 (43.8%) focused on disclosure only, using open disclosure.^27–31,40,42^ This involved an assigned liaison person contacting patients and families after incident, gathering data, convening a meeting for clinical staff to plan disclosure, determining its level of formality, facilitating a disclosure meeting, and apologizing. While it was suggested that maintaining contact played a part, the core focus was on the initial disclosure interaction, with less emphasis on what happened subsequently.

### Reconciliation

Only one intervention study (6.3%) focused on reconciliation alone, using the Improving Post-Event Analysis and Communication Together (IMPACT) tool.^39^ This involved using an interview guide to engage patients and families in organizational learning.

### Disclosure and Reconciliation

Eight intervention studies (50.0%) focused on both disclosure and reconciliation using 4 interventions comprising communication resolution programs (CRPs, n = 4, 25%),^34–36,38^ next-of-kin involvement (n = 2, 12.5%),^43,44^ the “disclosure, apology and offer” (DA&O) model (n = 1, 6.3%),^23^ and recognize, respond and resolve (3Rs, n = 1, 6.3%).^24^ Three intervention types (CRPs, DA&O, 3Rs) targeted reconciliation via financial compensation, and next-of kin involvement offered follow-up emotional support. Communication resolution programs and the DA&O model were designed to support disclosing and discussing incidents with patients and families, and proactively offering compensation where standards of care were not met. However, the DA&O model was largely aimed at staff, arguably assuming a passive approach of patients and families, and omitting their perspective within the study. 3Rs was a voluntary physician program to support the doctor-patient relationship after an incident via apology, explanation, and medical expense reimbursement. Next-of-kin involvement involved premeetings between doctors and legal professionals to prepare for meetings with next-of-kin, where issues related the incident were discussed and follow-up support was offered.

### Stakeholder Perspectives

Findings are organized according to the perspectives of 4 stakeholder groups: (1) patient and family, (2) healthcare professional, (3) nonclinical staff, and (4) legal staff. Studies gained perspectives from 1 (n = 14, 51.9%), 2 (n = 7, 25.9%), or 3 (n = 6, 22.2%) stakeholder groups. No single study considered the perspectives of all 4 groups (Table 4).

**TABLE 4:** Study Characteristics According to Stakeholder Group

Perspective	No. Studies Considering Their Perspective	Avg. No. Participants Bringing the Perspective Per Study	Total No. Participants Bringing the Perspective Across Studies*	Avg. Weighting of Their Perspective†
Patient/family	15	41.4	615	77.2%
Healthcare professionals	13	38.5	501	52.0%
Nonclinical staff	13	32.8	422	54.6%
Legal	5	9.0	45	21.5%

*In some instances, participants were involved in single studies multiple times, and the same data were reanalyzed across multiple studies. In both cases, participants were counted multiple times.

†This average is calculated based on the percentage of stakeholder representation within the total sample size of each included study.

### Patient and Family

Patients and families were represented within 15 studies (55.6%).^24–26,28,30,31,33,37–40,43,45,47,49^ The findings from these studies suggested that most patients and families valued being involved. However, investigations were complex events requiring staff sensitivity to a multitude of factors to avoid compounding harm. Patients and families were found to have wide-ranging needs and reported physical, financial, and/or emotional vulnerability, sometimes exacerbated by inadequate investigation processes. Presenting concerns included unclear expectations, inappropriate disclosure of unexpected outcomes, absent or insincere apologies, insufficient support, denying the opportunity to meet with staff, and delays. It was also found that some patients and families who felt involved in transparent investigation processes reported being less likely to pursue litigation, whereas others felt the need to fight for progress, using methods such as “threatening litigation.”^24^ Within maternity, studies reported parents articulating concern about information being withheld, not knowing how to be involved, information being inaccessible, and lacking understanding of formal processes.^45–48^ Needs included gaining information, telling their story, providing feedback, accessing emotional support, and achieving closure. In addition, some noted the importance of being offered advocacy and legal support. However, most preferred to be asked about their needs rather than them be assumed, as these varied.

### Healthcare Professional and Nonclinical Staff

Healthcare professionals who were directly involved or led investigations^23,25–27,29,30,32,38,41,42,46,48,49^ and nonclinical staff^23,25,29,30,32,34–38,42,44,49^ were each represented across 13 studies (48.1% each). Most healthcare professionals and nonclinical staff perceived that patient and family involvement could improve investigation quality, promote an open culture, and help ensure the safety of future care. However, it was made more difficult when multidisciplinary input was absent and workload and staff turnover were high. Staff also highlighted various needs including accessing adequate training to help prepare for, deliver and follow-up on investigations, and support from their organization, management, and colleagues. There was also a need to consider the complexities that litigation added. For instance, fears of inadequate legal protection, being individually blamed for systems failures and being unclear of the implications of apologizing, provided barriers to involvement for staff. While there seemed to be support for interventions specifically designed to actively encourage open disclosure and/or financial settlements,^23,24,34–36,38^ some fears from staff remained and were perceived to be difficult to culturally overcome.

### Legal Staff

Legal staff who acted on behalf of patients and/or healthcare organizations were represented in only 5 included studies (18.5%),^23,34–37^ 4 of which were conducted in the United States,^34–37^ which may reflect the insurance-based healthcare economy. Some legal representatives reportedly felt that proactive compensation resulted in higher proportions of harmed patients being compensated efficiently,^23^ and others found that it improved the timeliness that legal staff were notified of incidents, allowing them to be more proactive and collaborative.^34^ However, distance from the point of care meant that legal staff could not always get timely access to information,^34^ and others felt that the claims process did not meet patients’ emotional needs.^37^ Some lawyers suggested that in their experience, patients who felt that staff failed to engage with them were more likely to pursue legal action.^37^ However, because of limited representation (Table 4) and the way findings were reported in included studies, it was often difficult to disentangle the views of legal representatives from other stakeholders.

### Benefits and Challenges

Drawing upon findings from all eligible studies, a summary of the key benefits and challenges of patient and family involvement across these different perspectives are outlined.

### Benefits

#### Meeting a Moral Responsibility

Overall, for most stakeholder groups, involving patients and families in investigations was perceived to be a “moral responsibility.” It was reported to facilitate transparency, honesty, and standardization, acknowledge the importance of patient centeredness, and, in some cases, actively compensate those who experienced harm.^23,24,34–36,38^

#### Supporting Collaborative Improvement

Staff reported beliefs that involvement improved safety culture, fostered collaborative environments for shared learning, triggered reflexivity, and informed strategies to prevent incident reoccurrence.^22,23,25^ In maternity, staff also reported being able to gain clinically useful contextual information from parents not documented within medical notes.^45^ However, it was generally important for patients and families that words were congruent with action to indicate willingness to learn.

#### Rebuilding Therapeutic Relationships

Some studies reported that patients and families found coming together with staff close to the incident was positive and cathartic,^28–31^ whereas for others, this did not necessarily have to be with those directly involved.^33^ In addition, involvement reportedly increased the likelihood of feeling able to revisit the healthcare setting in the future for some^24,38^ and reduced the likelihood of escalation via other routes, such as litigation.^32,49^ Overall, patients and families reported perceptions that their involvement and offers of support helped foster a sense that their views were taken seriously, establish nonadversarial communication, and rebuild trust. However, support was needed both short and long term, but was often not available universally. Support seemed to be comparatively well established within maternity services, where included studies reported support for bereaved parents including information leaflets, expressions of condolences, and support from bereavement care midwifes, general practitioners, chaplaincy, lead clinicians, and bereavement counselors.^45–48^ Most spoke with one or more hospital worker after their child’s death and offers of emotional support tended to alleviate self-blame.^46^

### Challenges

#### Conflicting Views

Involvement was largely considered ideal practice but was not necessarily cohesive with instincts to protect the professional positions of staff, concerns of organizational reputational, or fears of litigation. Some reported hiding errors, disclosing as little as possible, or putting a positive spin on the truth.^26,42^ Managerial support for patient and family involvement varied, including disengagement, open unsupportiveness, and conflict within clinical teams regarding if and what to disclose and how to approach investigations and litigation.^35^ The importance placed on shielding staff directly involved from other staff, patients, and families was argued to sometimes exacerbate the problem and put staff in ethically compromising situations.^41^ Some staff also perceived that patient and family involvement may have limited use in cases such as suicide where the family did not witness the incident, and others felt that involvement may hamper rather than aid investigations through insufficient experience and knowledge.^25,44,49^

#### Inadequate Training and Resource

Involvement demanded variable and resource-intensive groundwork in collaboration with multidisciplinary stakeholders.^29^ Overall, tasks were made difficult because of high workloads, limited allocated time within job plans, staff turnover, unclear remits, and unmet training needs. Some called for investment in medical education, robust coaching models, emotional support, and practical guidance for disclosure and involvement, and others felt that training needed to be tailored to local culture.^35^

#### Sensitivity to Trauma and Grief

Beyond clinical aspects of incident investigations, considering emotional responses was found to be of significant importance across studies. In maternity, staff faced difficulties including managing parents with unanswerable and unexpected questioning, anger and vulnerability, and demands of additional investigations that were not possible.^46^ Staff also discussed reactions such as guilt, self-blame, fear, sleeplessness, and anxiety, which they felt uncomfortable or unprofessional to disclose.^26^

#### Tokenistic Approach

Some patients and families felt a tokenistic approach to involvement was taken, such as inviting involvement once everything had been decided, or narrowly focusing investigations and reports. Within maternity, most parents valued continuity of care and wanted to meet with staff, but this was not always possible. In mental healthcare, there was little policy focus on listening to families and limited breadth and depth regarding involvement opportunities.^49^ Some also highlighted challenges with considering who to involve from larger families in investigations of suicide.^49^

#### Flexible and Personalized

Staff faced challenges in terms of determining how to engage with patients and families, who should initiate meetings and who should facilitate them. Some felt that each incident needed an individual to take the lead, streamline processes, and build trusting relationships, but there was not always capacity to provide a “single point of contact.”^45^ In addition, a range of complex adjustments that needed to be considered based on individual need were highlighted, including meeting location, language, cultural factors, meeting attendees, whether patients and families wanted or were able to be involved, and the method and timing of communication. For some, formal face-to-face interaction was considered authentic and respectful, whereas others preferred less intrusive methods that gave emotional space to process information.^38,39^ In maternity, most formalized follow-up occurred within 3 months, which was preferable for the majority, but felt too soon for some, and others did not want to meet at all.^47^ Similarly, in mental healthcare, organizational timelines did not always allow for grieving, recuperation, or not wanting to be involved immediately, yet stopped other families from moving on.^49^

### Potential Solutions to Support Involvement

Across included literature, there were a number of suggested solutions that might support greater involvement in incident investigations. Some studies reported that clarity of roles and responsibilities helped reduce miscommunication and streamline processes, which was sometimes easier in smaller organizations and where it became part of a well-established clinical governance system.^35,41^ In addition, targeted training and enhancing skills relating to involvement helped activities such as disclosure to be viewed as a moral and professional duty,^27^ supported by processes such as learning from experienced staff, informal support from colleagues, and formal support from management and project champions. Mental healthcare policies helped guide staff in terms roles and responsibilities and how to report information, inform families of suicide, give feedback to families, follow up, and evaluate improvements and how to deal with refusal of family involvement and privacy issues.^49^

## DISCUSSION

Our review considers what is currently known about the experiences, values, and needs of key stakeholders when patient and families are involved in serious incident investigations, and the benefits and challenges of their involvement. It also explores the identified potential solutions to support their involvement. The included papers suggest that while stakeholders may experience wide-ranging challenges when patients and families are involved in investigations, all stakeholders widely perceive involvement to be ethically and instrumentally beneficial to improve transparency, rebuild therapeutic relationships, collaboratively enhance organizational learning, and help allow patients and families to access further support. Involvement is also reported to be made easier for staff when they are guided by policy, and structured processes help ensure that further harm is not experienced by patients and families as a result of the processes after an incident. However, the collective findings also suggest that involvement is a highly complex process, requiring sensitive management, with this complexity often incompatible with organizational culture. One significant barrier to involvement is the fear about litigation processes. The potential solutions to support involvement include enhancing the clarity of roles and responsibilities within investigations, adequately training staff, and providing long- and short-term support to key stakeholders.

To avoid being perceived as tokenistic, a flexible and personal approach to involvement was required in practice, with sensitivity to trauma and grief. This highlights the importance of attending to both clinical and emotional aspects of care and considering the needs and perspectives of all stakeholders when aiming to meaningfully involve patients and families in serious incident investigations. It is also important to acknowledge that these needs may conflict.

Drawing together the learning from this review, we have developed a number of specific recommendations for designers of incident response systems, to support the involvement of patients and families in investigations (Box 1).

Box 1 Recommendations for designers of investigation processes

- Ensure systems are flexible and support individualized patient and family responses by actively listening with empathy for trauma, fostering a sense of trust and transparency, clearly communicating realistic timelines, and establishing nonadversarial communication;
- Systems should attempt to address the range of needs that arise, in addition to “repairing” the original harm;
- Targeted training should be provided to staff seeking to involve patients and families in incident investigations;
- Specific service provision should be made for patients and families, and healthcare staff involved in serious incidents and their investigation, to support the range of physical, financial, and/or emotional impacts experienced;
- Organizations should consider implementing policies and rolling out interventions that support processes of reconciliation, restoration, and rebuilding of trust;
- Organizations should identify and address culturally embedded fears surrounding involvement;
- Organizations need to resource investigation systems adequately to accommodate these recommendations.

While this review provides valuable insights for policymakers, healthcare organizations, staff, patients, and families on how to meaningfully incorporate patient and family involvement, there are significant gaps within the evidence. For example, most studies focused on the disclosure element of serious incident investigations only. Zimmerman and Amori^50^ posed that a passive role in this element of investigations alone was insufficient and that patients and families need to be engaged with in an increasingly transparent manner and play a greater role in the process of change. According to the Involvement Matrix proposed by Smits et al,^51^ much of the included literature falls within the “lesser” involvement domains, instead of viewing patients and families as “active partners” or “decision makers” within investigations. Moving forward, it is important to consider the extent to which healthcare services continue to limit patient and family involvement in the processes following healthcare harm.

In addition, the current literature omits the consideration of stakeholder groups and how learning can be applied across settings. For instance, the perspectives of nonclinical staff and legal representatives in maternity services, which is currently a topic of considerable focus for policy makers, managers, and healthcare professionals within the United Kingdom. Furthermore, the perspectives of legal representatives in mental healthcare and single studies that consider all perspectives are largely absent from included studies. As much of the research has been based in acute care and hospital setting, greater attention needs to be paid to the phenomenon of patient and family involvement in other healthcare settings, with a need to develop interventions that can be adapted to specific contexts, such as mental health and maternity, alongside the more general learning from hospital care where incident type is more diverse.

Finally, given that research focused on CRPs^34–36,38^ covered the most ground in terms of the perspectives sought and considering both disclosure and reconciliation, adapting this intervention for mental health, maternity settings, and/or in different countries and healthcare systems could help address this gap. Other fruitful research areas would include the exploration of how interventions that actively offer compensation (such as CRPs, DA&O, 3Rs) might be applies to nonpaid healthcare systems, such as the United Kingdom.

### Limitations

Methodologically, this review searched only 3 databases, and therefore, relevant literature may have been omitted. However, Connected Papers was used to reduce the risk of missing key articles. Because of the nature of the research area and aims, it was appropriate that the review adopted a scoping approach, did not quality assess, and identified qualitative studies only. However, we acknowledge that using such methods may have offered different perspectives and determined causal relationships. Finally, there was no inclusion of studies outside of western cultures, which may be a product of the lack of research and/or methodology.

### Conclusions

Our review provides valuable insights to ensure that patient and family involvement in serious incident investigations considers both the clinical and emotional aspects of care, is meaningful for all key stakeholders, and avoids compounding harm. However, significant gaps within the literature remain.
